# The Structural Diversity of Deoxyribozymes

**DOI:** 10.3390/molecules15096269

**Published:** 2010-09-06

**Authors:** Simon A. McManus, Yingfu Li

**Affiliations:** 1 Department of Biochemistry and Biomedical Sciences, McMaster University, 1200 Main Street West, Hamilton, Ontario L8N 3Z5, Canada; E-Mail: mcmanusa@mcmaster.ca; 2 Department of Chemistry and Chemical Biology, McMaster University, 1200 Main Street West, Hamilton, Ontario L8N 3Z5, Canada

**Keywords:** deoxyribozyme, DNAzyme, catalytic DNA, *in vitro* selection, functional nucleic acid

## Abstract

When not constrained to long double-helical arrangements, DNA is capable of forming structural arrangements that enable specific sequences to perform functions such as binding and catalysis under defined conditions. Through a process called *in vitro* selection, numerous catalytic DNAs, known as deoxyribozymes or DNAzymes, have been isolated. Many of these molecules have the potential to act as therapeutic agents and diagnostic tools. As such, a better understanding of the structural arrangements present in these functional DNAs will aid further efforts in the development and optimization of these useful molecules. Structural characterization of several deoxyribozymes through mutagenesis, *in vitro* re-selection, chemical probing and circular dichroism has revealed many distinct and elaborate structural classes. Deoxyribozymes have been found to contain diverse structural elements including helical junctions, pseudoknots, triplexes, and guanine quadruplexes. Some of these studies have further shown the repeated isolation of similar structural motifs in independent selection experiments for the same type of chemical reaction, suggesting that some structural motifs are well suited for catalyzing a specific chemical reaction. To investigate the extent of structural diversity possible in deoxyribozymes, a group of kinase deoxyribozymes have been extensively characterized. Such studies have discovered some interesting structural features of these DNAzymes while revealing some novel DNA structures.

## 1. Introduction

Nucleic acids were once thought to be used solely for the storage and transfer of genetic information in living cells, with proteins performing other cellular functions such as catalysis. The discovery of an RNA capable of self-splicing changed this view and indicated that at least one of the nucleic acids, RNA, was capable of catalyzing chemical reactions [1]. Following this, several other natural catalytic RNA molecules, termed ribozymes, were found [2,3,4,5,6]. It is now widely believed that before the emergence of proteins, RNA performed the majority of cellular functions [7]. This notion has been further supported by the isolation of numerous artificial ribozymes through a process called *in vitro* selection, demonstrating that RNA is capable of catalyzing a broad range of chemical reactions (reviewed in [8,9]).

Following the discovery of these natural and artificial ribozymes, researchers pondered whether DNA was also capable of catalysis. DNA is most commonly found in long helical duplexes. In this highly constrained form, it is unlikely that DNA can arrange itself into a structure that can form interactions with a substrate and catalyze a reaction. Single-stranded DNA on the other hand, is much less structurally constrained and has properties similar to RNA. Through *in vitro* selection, Breaker and Joyce were able to isolate the first catalytic DNA molecule that was able to cleave an RNA linkage within a DNA substrate in the presence of Pb^2+^, demonstrating that DNA could indeed act as a catalyst [10]. In subsequent years, several more catalytic DNA molecules, called deoxyribozymes or DNAzymes, have been isolated to catalyze other biologically relevant chemical reactions (reviewed in [11,12,13]), such as DNA ligation [14,15], RNA ligation and branching [16,17,18], DNA phosphorylation [19,20], DNA adenylation [21], DNA cleavage [22], DNA hydrolysis [23], porphyrin metalation [24], thymine dimer repair [25], nucleopeptide bond formation [26], and carbon-carbon bond formation [27]. Despite these many examples of DNA-mediated catalysis, it is noteworthy that most of the DNAzymes isolated to date bind and process nucleic acid-containing substrates. Since DNA has an inherent ability to bind these nucleic acid targets, there is a lingering question of whether DNA is capable of catalyzing a broader range of chemical reactions that do not involve nucleotides or nucleic acids as substrates. Although there is no doubt that more such DNAzymes will be isolated in the future *in vitro* selection experiments, it is certainly beneficial to examine fundamental properties of existing deoxyribozymes. Examining the structures used by current deoxyribozymes is a good starting point.

**Scheme 1:**
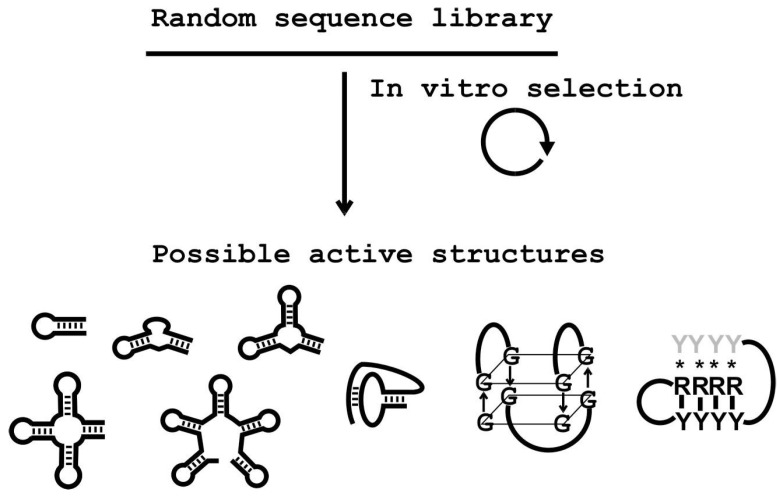
Deoxyribozymes isolated through *in vitro* selection have been shown to use many different arrangements in their active structures, such as Watson-Crick helices and higher-order structures containing guanine quadruplex and triple helix motifs.

The terminology of DNA structure is hierarchical. Primary structure is the nucleotide sequence, which can be readily obtained for isolated deoxyribozymes by DNA sequencing [28,29]. Secondary structure of nucleic acids is defined as double-helical interactions formed through Watson-Crick base-pairs. Predictions for DNA secondary structure can be obtained through folding algorithms, once the primary sequence is known [30] and tested through mutagenesis analysis. Tertiary structures are generally regarded as interactions other than Watson-Crick base-pairing, such as triplex interactions [31], guanine quadruplexes [32] and i-motifs [33]. Predicting the types of structures that will arise from an *in vitro* selection is difficult as these selections typically begin with randomized libraries. The usual process is to perform the selection until the desired level of activity is seen, and sequence the population. Structural characterization is then performed on representatives from the most abundant sequence classes (Scheme 1). Currently, the amount of structural information available for deoxyribozymes depends on whether the deoxyribozymes contain structures that are predominantly secondary or tertiary. On the one hand, a good amount of secondary structural information is available for most deoxyribozymes. In fact, most initial reports of new deoxyribozymes or follow-up studies include secondary structural models. This is due to the ease and low cost of synthesizing mutant constructs of a particular deoxyribozyme to test the existence of Watson-Crick base-pairing regions (for example, mutating a predicted A-T pair to a C-G pair to test whether base-pairing is required for activity). In addition, *in vitro* re-selection experiments using a partially mutated library can reveal useful information on the potential base-paring interactions within a given deoxyribozyme.

Conversely, much less information has been obtained on the tertiary structures of most deoxyribozymes. This is due largely to the fact that higher resolution techniques, such as X-ray crystallography and NMR, have so far not produced any models of deoxyribozymes in their active structures. Despite this drawback, some tertiary structural information has been obtained for certain deoxyribozymes through various chemical footprinting techniques, such as methylation interference which can identify guanine residues whose N7 position is involved in a non-Watson-Crick interaction.

This review intends to examine structural diversity of known deoxyribozymes through the use of specific examples to show how deoxyribozymes can use common or different structural arrangements as the structural foundation to carry out relevant catalytic functions. The last section will deal with our own research in which a model reaction system is used to show the level of structural diversity observed within a specific category of deoxyribozymes that catalyze DNA phosphorylation.

## 2. The Two Binding-Arm Catalytic Core Motif

**Figure 1:**
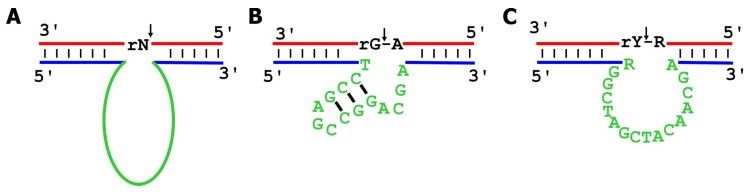
RNA-cleaving deoxyribozymes using a two-binding arm motif. (a) A general structural framework for RNA-cleaving deoxyribozymes that use the two binding arm motif. The deoxyribozyme contains two regions called binding arms (shown in blue) that base-pair to the substrate (shown in red). The RNA cleavage site (indicated with an arrow) is between the two base-paired regions. The catalytic core (shown in green) is opposite the cleavage site. rN represents the ribonucleotide at the cleavage site. (B) Secondary structural model for the 8-17 deoxyribozyme. (C) Secondary structural model for the 10-23 deoxyribozyme. At the cleavage site, rY can be rU or rC, and R can be G or A.

Deoxyribozymes that cleave RNA have been subject to extensive studies. These deoxyribozymes are of great appeal as mRNA cleavage (and subsequent gene expression reductions) has many research and therapeutic implications. Consequently, numerous *in vitro* selection experiments have been conducted, resulting in the isolation of many RNA-cleaving DNAzymes [10,34,35,36,37,38,39,40,41,42] (reviewed in [43]). Due to large numbers of available sequences, this would seem like a logical place to start looking for structural diversity between different deoxyribozymes. However, in terms of structural diversity, most of these RNA-cleaving deoxyribozymes seem to share a common structural arrangement categorized by a small catalytic domain flanked by two regions that bind the RNA substrate through Watson-Crick base-pairing (see the model shown in Figure 1a). 

Even with this general structural framework, there are considerable structural variations within the catalytic cores of different RNA-cleaving deoxyribozymes. A good example is the structural variations between 10-23 and 8-17. These two deoxyribozymes were initially discovered in the same *in vitro* selection experiment [37]. Extensive structural studies on 8-17 have shown that its small catalytic core contains a three base-pair stem (Figure 1b) that can accept many sequence variations [44,45]. In contrast, 10-23’s catalytic core has not been found to contain a defined secondary structure (Figure 1c) and the bases on most nucleotides within its catalytic core are intolerant to mutations [46,47]. This suggests that these bases may be involved in tertiary interactions that allow the catalytic domain to fold into its active conformation to execute catalysis. The structural variations within these two RNA-cleaving deoxyribozymes that share a common framework of two binding arms demonstrate that deoxyribozymes are capable of exploring some levels of structural diversity to achieve the catalysis even for a relatively simple chemical reaction such as RNA cleavage. However, a relatively easy chemical transformation like RNA cleavage (which has a detectable uncatalyzed reaction rate) may simply favor the isolation of small and simple structures that exist in a random-sequence DNA library by the *in vitro* selection technique with which all deoxyribozymes have been obtained to date. Evidence for this thinking comes from the fact that the structurally simple 8-17 deoxyribozyme has been repeatedly isolated in several *in vitro* selection experiments by different laboratories using different DNA libraries [36,37,45,48,49]. The ability of this small catalytic DNA motif to ‘hijack’ a selection may be the reason that more complex structures are not found in these experiments. For this consideration, it may be necessary to examine deoxyribozymes that catalyze more demanding chemical reactions in order to assess if deoxyribozymes can exhibit a higher degree of structural diversity (*i.e*. different structural scaffolds).

## 3. Different Secondary Structural Arrangements

**Figure 2:**
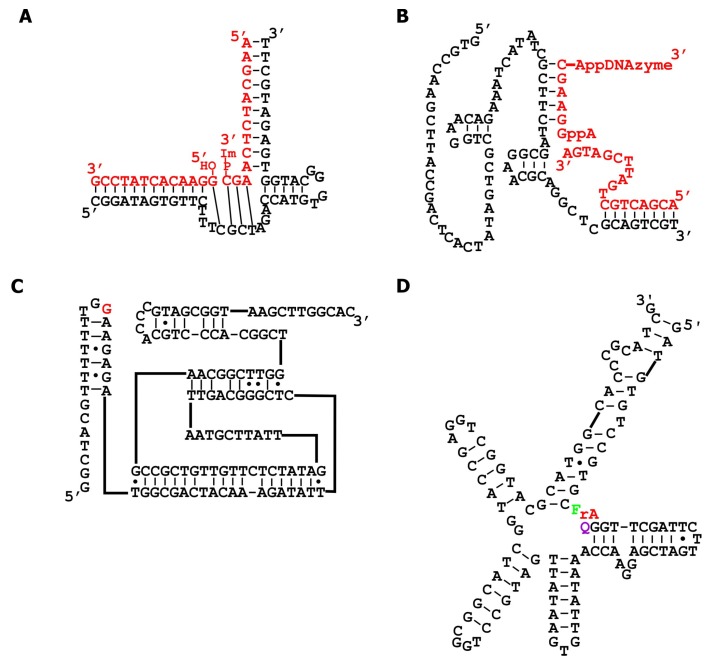
Deoxyribozymes with different secondary structural arrangements. (A) Structural model of the E47 ligase deoxyribozyme. Substrates are shown in red. OH represents a 5′ hydroxyl and P-Im represents a 3′ phophorimidazolide. Solid lines represent Watson-Crick base-pair interactions. (B) Secondary structural model of the L78 DNA ligase deoxyribozyme. 5′,5′ pyrophosphate cap is represented by ppA. (C) Secondary structural model of the 10-28 N-glycosylase deoxyribozyme. Site of glycosylation is shown with a red G. Circles represent G-T wobble base-pairs. (D) Secondary structural model of 5J-A28, an RNA-cleaving, fluorescence-generating deoxyribozyme. F and Q represent a fluorescein- and a DABCYL-containing deoxythymidine, respectively. rA denotes adenine ribonucleotide.

Although the two binding arm motif seems to be the favored structural arrangement for RNA-cleaving deoxyribozymes as well as some deoxyribozymes that catalyze the reverse RNA ligation reaction [16], other systems appear to use different structural arrangements. In 1995, shortly after the discovery of the first RNA-cleaving deoxyribozyme, a deoxyribozyme for joining two DNA oligonucleotides together was isolated [14]. This deoxyribozyme, named E47, ligates the 5′ hydroxyl of one DNA substrate to a second, activated DNA molecule, creating a phosphodiester bond between them. Its secondary structure (as shown in Figure 2a) contains a binding arm for one substrate at the 3′ end of the deoxyribozyme with a TTT bulge near the ligation site while the 5′ end binds the other substrate. Interestingly, this 5′ portion also contains an internal stem, creating a three-way helical junction. With this structure the nucleobases across from the ligation site are involved in base-pairing interactions with the substrate. Therefore, this DNA-ligating deoxyribozyme must be using residues from distal regions to perform catalysis.

Almost ten years later another DNA-ligating deoxyribozyme was discovered that also contained a unique structural arrangement as shown in Figure 2b [15]. In this case the deoxyribozyme mimics the second reaction catalyzed by T4 DNA ligase, the ligation of adenylated DNA (AppDNA) to an acceptor DNA oligonucleotide. The AppDNA substrate is held in place through six base-pairs with the deoxyribozyme, while the other substrate was found to have eight base-pair interactions with the deoxyribozyme. Notably, this secondary substrate contains eleven unpaired nucleotides adjacent to the ligation site. This suggests that the deoxyribozyme uses base-pairing in combination with other unidentified interactions to bind this substrate and place it in the correct orientation for catalysis. Also distinct to this deoxyribozyme in relation to the previously discussed deoxyribozymes is the large amount of essential sequence outside the substrate-binding region on the 5′ end of the deoxyribozyme. This sequence element is probably involved in undefined tertiary interactions, adding to the complexity of the structure.

Another deoxyribozyme with a distinct secondary structural arrangement is the 10-28 deoxyribozyme that catalyzes N-glycosylation of a specific guanine residue within a DNA substrate [50]. This deoxyribozyme has arguably the most complex secondary structure of known deoxyribozymes. As shown in Figure 2c, the model contains two stem-loops at the 5′ and 3′ ends, and two stems in the center of the deoxyribozyme. The internal stems are entwined in a type of pseudoknot with part of one stem being in the loop at the end of the second stem and vice versa. The fact that this deoxyribozyme can use such a complex arrangement of interconnected double-helical interactions illustrates that deoxyribozymes can have substantial structural complexity, at least on the secondary structural level.

Deoxyribozymes have also been isolated that utilize other complex helical arrangements. In one study, an *in vitro* re-selection experiment was performed to increase the efficiency of an RNA-cleaving fluorescence-generating deoxyribozyme that utilized a three-way helical junction in its active structure [51]. After sequencing the population in the fifth round of the re-selection, two classes were observed that utilized an active structure containing a five-way helical junction arranged in a star-like pattern. With more selection rounds under stringent selection conditions, the star-structured sequences out-competed the original three-way junction motif and became the dominant class, suggesting that this complex structure is more efficient at performing this particular reaction. 

## 4. Deoxyribozymes with Known Tertiary Interactions

**Figure 3:**
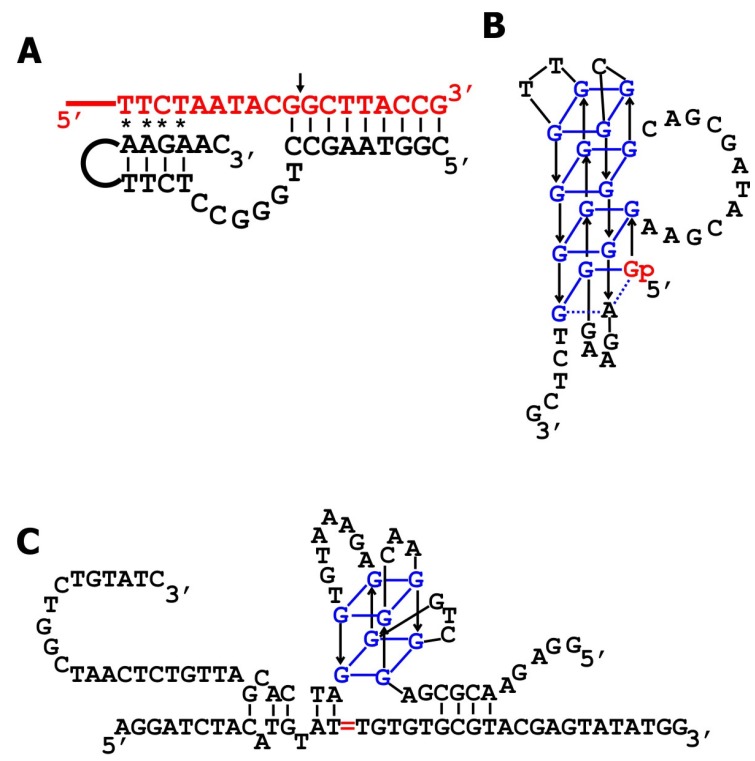
Deoxyribozymes with tertiary interactions. (A) Structural model of a DNA-cleaving deoxyribozyme utilizing a triplex structure. The substrate is shown in red and the cleavage site indicated by an arrow. (B) Structural model of the class I self-capping deoxyribozyme containing a multi-tiered guanine quadruplex. Guanines involved in quadruplex interactions are shown in blue. Gp represents a 5′ phosphoryated guanine. (C) Structural model for the UV1C thymine dimer repair deoxyribozyme containing a two-tiered guanine quadruplex. Red parallel lines represent the thymine dimer (the thymines within the dimer are not connected by a phosphodiester bond). The quadruplex is thought to act as a light-harvesting antenna facilitating the repair of the T-T dimer.

It has long been known that DNA is capable of forming structures other than the Watson-Crick double helix commonly seen in biological systems. Two prominent tertiary structures are guanine quadruplexes and triple helices. These structures have also been found in the active structures of deoxyribozymes. Triple-stranded DNA structures are formed by stretches of pyrimidine-purine-pyrimidine base triples such as T**^.^**AT and C^+^**^.^**GC [31,52,53]. Carmi *et al*. found such a triple helix to be present in the active structure of a deoxyribozyme that cleaves a DNA substrate [22]. During secondary structural analysis, they noticed that a string of unpaired pyrimidines in the substrate had the correct sequence to form a triple-helical complex with a polypurine-polypyrimidine stem in the deoxyribozyme, as seen in Figure 3a. Through alterations of bases they were able to show that the deoxyribozyme was active with different variations of the triple helix, but became inactive if the triplex was disrupted, showing that this DNA-cleaving deoxyribozyme indeed contains a triplex in its active structure.

Guanine quartets consist of four guanines arranged in a planar square arrangement with two hydrogen bonds formed between each adjacent guanine [54,55]. Stacked quartets called quadruplexes have been found to be very stable and have been isolated in telomeric sequences at the end of chromosomes, as well as in the promoter regions of certain genes [32,56,57,58]. Investigations have shown that several deoxyribozymes may contain guanine quadruplexes in their active structures [19,21,25,59]. Some examples of guanine quadruplex-containing structural models are shown in Figure 3b and 3c. One interesting example is a thymine dimer repair deoxyribozyme with a two-tiered guanine quadruplex directly across from the dimer repair site. It is hypothesized that apart from a structural role this quadruplex may also act as an antenna to harness light energy used to repair the dimer. If this is the case, it demonstrates that deoxyribozymes may be capable of using different structural folds to alter their properties possibly allowing DNA to catalyze complex reactions. 

Intriguingly, while several DNAzymes have been shown to contain quadruplexes in their active structures, quadruplexes are not observed in ribozymes. The known classes of natural ribozymes, whose structures have been intensively studied (reviewed in [60]), use extensive Watson-Crick base-pairing in their structural scaffolds. Artificial ribozymes isolated by *in vitro* selection have also been found to contain primarily structural scaffolds built with Watson-Crick helical elements [61]. Thus, it appears that deoxyribozymes are more prone to incorporating quadruplex arrangements in their active structures than ribozymes. The reasons for this difference in structural preference are unclear at present. Quadruplexes have been rationally designed into ribozymes to act as regulatory components [62,63], and a quadruplex-containing DNAzyme has been converted into a ribozyme [64], showing that quadruplexes can be utilized by RNA catalysts. The presence of quadruplexes in many DNAzyme structures may reflect the fact that a higher number of strand conformations are available for DNA quadruplexes than for RNA quadruplexes. RNA quadruplex-forming sequences have been shown to fold exclusively into all-parallel conformations due to orientation of the ribose pucker [65], while DNA quadruplexes have been shown to adopt parallel, antiparallel and mixed strand orientations [66]. This increase in the number of possible conformations may translate into more functional (catalytic) quadruplex scaffolds being present in a random-sequence DNA pool than an RNA pool. 

## 5. Deoxyribozymes that Function at Low pH

The DNAzyme structures discussed thus far are based on interactions that are prominent at neutral pH. At low pH (high acidity), potential hydrogen-bond acceptors, such as the N3 of cytidine (pKa = 3.5) and the N1 of adenosine (pKa = 4.2), become protonated, which disfavors the formation of Watson-Crick base-pairs. With these protonations, different interactions, such as base-pairs involving protonated cytosines (C^+.^C), adenines (A^+.^ A^+^) or triple-base interactions (C^+.^CG) become favorable [67]. 

To test whether DNAzymes will form different types of structures in acidic conditions, a series of studies were carried out by our group to isolate and characterize RNA-cleaving DNAzymes that function at a range of acidic pH. Several rounds of *in vitro* selection were performed at pH 4, and the active population was subsequently subjected to five parallel selections performed at pH 3, 4, 5, 6 and 7 [68]. Several catalytically active sequences were found in each reaction pool. Overall, most sequences were found in only one pH population and no common sequences were found in all five pools. The lack of a common sequence across the five populations suggests that different structures are necessary to perform RNA-cleavage at different pHs. Structural characterization revealed that the DNAzymes functioning near neutral pH utilized Watson-Crick base-pairing, while those functional at lower pH used different interactions. DNAzyme pH7DZ1, functional at pH 7, was found to contain two Watson-Crick base-paired regions in its active structure [68]. Similarly, pH6DZ1 which displayed optimal activity at pH 6, was also found to be highly helical, containing a four-way junction [69]. In contrast, pH5DZ1, a DNAzyme that was functional at pH 5, was not found to contain any Watson-Crick base-pairing interactions after exhaustive mutational analysis [70]. Similar results were obtained with DNAzymes that function at pH 4 (pH4DZ1) [71] and pH 3 (pH3DZ1) [72], suggesting that these DNAzymes must be folding into different types of structures mediated by non-Watson-Crick interactions. Chemical footprinting analysis also suggested the presence of different structural interactions in the low pH DNAzymes. For instance, methylation of certain guanine residues was found to lead to an increase in activity, contrasting with the usual observation of methylation interfering with DNAzyme activity. High-resolution study of these deoxyribozymes will be of particular value as it can reveal the exact nature of these interactions and show which structural folds are preferable to DNAzymes that function at low pH. 

## 6. Kinase Deoxyribozymes as a Model System to Study Structural Diversity

In the preceding sections we have discussed the structures of deoxyribozymes catalyzing a wide range of reactions or under different conditions such as pH, and shown many deoxyribozymes to use distinct structural arrangements to catalyze these reactions. Our group has also examined the structural diversity of a group of deoxyribozymes for DNA phosphorylation. The chemical reaction investigated is the transfer of a γ-phosphate from a nucleoside 5′-triphosphate (such as ATP) to the 5′ end of a DNA substrate and the related deoxyribozymes are called kinase deoxyribozymes (or self-phosphorylating deoxyribozymes). 

A group of kinase deoxyribozymes were first isolated by Li and Breaker in a study to investigate deoxyribozyme substrate specificity [19]. A second study was performed by our group to derive efficient deoxyribozymes with different divalent metal ion co-factor requirements [20]. We decided to use some of the kinase deoxyribozymes from this second study as a model system to examine structural diversity. We chose five unique sequences that were observed multiple times in the pool from the sixteenth round of a sub-selection performed under stringent reaction conditions. In this selection, reaction time was progressively decreased in order to obtain efficient catalysts with fast reaction rates. These sequences were named Deoxyriboyzme kinase 1 to 5 (Dk1 to Dk5) in order of abundance. The secondary structures of the two most abundant sequences Dk1 (ATP-dependent) and Dk2 (GTP-dependent) were elucidated and showed both structural similarities and differences [73]. Both deoxyribozymes appear to have a central stem-loop motif that acts to anchor two arms that are responsible for substrate binding and catalysis. This stem-loop motif was shown to be purely structural and not involved in catalysis, as its base-pair sequence could be altered without loss of activity. In fact, it was found that the stem-loops from each deoxyribozyme could be interchanged between the two deoxyribozymes without change in function. Further characterization revealed that Dk2 has other base-pair interactions in its active structure, while Dk1 does not. The methylation interference patterns for the two deoxyribozymes were also different, indicating they use differing tertiary interactions in order to fold into their respective active structures. 

**Figure 4:**
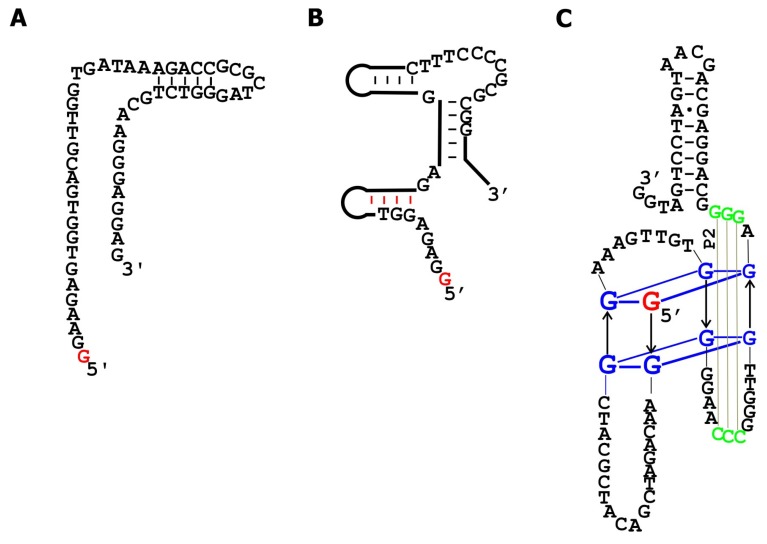
Structure diversity of kinase (self-phosphorylating) deoxyribozymes. (A) Structural model of Dk1, an ATP-utilizing kinase deoxyribozyme. Dk1 contains a central stem flanked by two unstructured regions. The site of self-phosphorylation is shown in red. (B) Secondary structural model for GTP-utilizing kinase deoxyribozymes Dk2, Dk3, and Dk4. Red lines indicate base-pairs that are present in Dk3 and Dk4 but not Dk2. (C) Structural model of Dk5, an ATP and GTP utilizing kinase deoxyribozyme. The structure contains a two-tiered guanine quadruplex shown in blue. A three base-pair stem (shown in green) forms a pseudoknot interaction with the quadruplex.

Dk3 and Dk4 appeared to share a common secondary structure upon first inspection. Structural analysis revealed that they did indeed have a similar structural arrangement containing three stem-loops (Figure 4) [74]. In addition, sequences in the unpaired 5′ region, as well as three junctions between the stems (one being 14 nucleotides long), were nearly identical for both deoxyribozymes. Intrigued by this similarity we re-examined previously studied kinase deoxyribozymes to determine whether they also contain the common sequence elements observed with Dk3 and Dk4. This led to the revelation that Dk2 has a structural arrangement that is very similar to Dk3 and Dk4: all three deoxyribozymes contain a common, stem − 14-nucleotide junction − stem motif. Interestingly, this conserved junction sequence was located in different positions in the sequences of Dk2, Dk3 and Dk4. Because these deoxyribozymes were selected based on their size, the different location of this same sequence in Dk2-4 suggests that they are not evolutionarily related and arose independently from the original random-sequence pool. The multiple independent occurrences of this structural motif suggests that it may represent the easiest or the most efficient solution to DNA self-phosphorylation with the use of GTP as the phosphate source, similar to the case that 8-17 is the simplest structural solution for DNA catalyzed RNA cleavage. 

Characterization of Dk5 revealed that this deoxyribozyme is peculiar: it is capable of using both ATP and GTP as the phosphorylation substrate and nonselective for divalent metal-ion cofactor, using Ca^2+^, Cu^2+^, Mg^2+^, or Mn^2+^. For comparison, Dk1-4 are all Mn^2+^-dependant and use a specific NTP substrate (either ATP or GTP). These distinct characteristics of Dk5 suggest that this deoxyribozyme may have a unique structural arrangement. Structural characterization of Dk5 also reveals that this deoxyribozyme adopts a very distinctive active structure [75] containing a two-tiered guanine quadruplex and two Watson-Crick stem-loop regions (Figure 4). More interestingly, two of guanines involved in the quadruplex are in the loop of one of the stem-loops, creating a novel pseudoknot arrangement. Circular dichroism analysis on mutant constructs indicates that the presence of this stem is essential for quadruplex formation. To our knowledge, this is the only reported case of a guanine quadruplex and a double-helical region being involved so closely in the same structure. 

## 7. Conclusions 

Through the examples shown above, it is apparent that DNA, despite its lower level of chemical diversity as opposed to proteins, is still capable of using many different structural arrangements to “create” deoxyribozymes. This is true both on the secondary structure level (with different deoxyribozymes using different double-helical arrangements) and on the tertiary structure level (with some deoxyribozymes employing triplex and guanine quadruplex motifs in their active structures). Our study of structural diversity within deoxyribozymes catalyzing the same chemical reaction, using kinase deoxyribozymes as a model system, has revealed three distinct structural arrangements from a group of five deoxyribozymes. The revelation that deoxyribozymes adopt various complex structural arrangements to carry out catalysis on nucleic acid-based substrates or nucleic acid-containing substrates suggests that DNA may also be capable of “creating” more complex structures needed to perform more difficult or more diverse reactions that involve non-nucleic acid substrates. 

To increase the likelihood of isolating deoxyribozymes with large complex active structures, it may be necessary to change the way that these deoxyribozymes are selected. The libraries that have been used to select for all known deoxyribozymes have contained relatively short random-sequence regions (40 to 100 nucleotides). While these libraries have been very effective in isolating deoxyribozymes with small motifs that exhibit some level of structural complexity, undoubtedly more complex motifs will have to be derived from DNA libraries containing much larger random-sequence domains. In a study of the effect of random-sequence length on ribozyme selection, it was found that larger structural motifs were much more readily found in libraries with larger random regions [76]. Computational analysis suggests that completely randomized RNA libraries mainly contain sequences that fold into simple structures such as low-branching and linear folds, while more complex structures such as higher-order helical junctions are underrepresented [77]. As DNA has similar folding properties as RNA, these conclusions can also be applied to random-sequence DNA pools. In order to obtain libraries that are not biased towards simple structures, it may be necessary to incorporate some rationality into the library design at the start of an *in vitro* selection experiment, such as designing libraries with a mixture of pre-engineered structural folds. With such modifications to the powerful *in vitro* selection technique, it should be possible to isolate larger and better deoxyribozymes with very complex structures and increase the repertoire of reactions beyond what has been currently demonstrated.
